# 

**Published:** 1907-09

**Authors:** 


Sixty-third Year.

VOL. LXIII. SEPTEMBER, 1907. NO. 2

### BUFFALO MEDICAL JOURNAL

Established 1845 by AUSTIN FLINT M. D.

LIBRARY.

SURGEON GENERAL'S OFFICE.

SEP. 8. 1907

EDITOR:

WILLIAM WARREN POTTER, M. D.

ASSISTANT EDITORS:
WILLIAM C. KRAUSS, M. D. NELSON W. WILSON, M. D.

ASSOCIATE EDITORS:
JAMES WRIGHT PUTNAM, M. D. ERNEST WENDE, M. D.
JOHN PARMENTER, M. D. JOHN A. MILLER, PH. D.
MAUD J. FRYE, M. D.

